# Synergistic effects of dendrosomal nanocurcumin and oxaliplatin on oncogenic properties of ovarian cancer cell lines by down-expression of MMPs

**DOI:** 10.1186/s40659-023-00412-x

**Published:** 2023-01-20

**Authors:** Elahe Seyed Hosseini, Marziyeh Alizadeh Zarei, Hossein Tarrahimofrad, Javad Zamani, Hamed Haddad Kashani, Ejaz Ahmad, Hossein Nikzad

**Affiliations:** 1 Gametogenesis Research Center, Institute for Basic Sciences, Kashan University of Medical Science, Kashan, Iran; 2 Anatomical Sciences Research Center, Institute for Basic Sciences, Kashan University of Medical Sciences, Kashan, Iran; 3 Department of Animal Biotechnology, National Institute of Genetic Engineering and Biotechnology, Tehran, Iran; 4 Department of Plant Biotechnology, National Institute of Genetic Engineering and Biotechnology, Tehran, Iran; 5 Department of Pathology, Michigan Medicine, University of Michigan, Ann Arbor, MI 48109 USA

**Keywords:** Ovarian cancer, Oxaliplatin, Curcumin, Dendrosomal nanocurcumin, Apoptosis, Metastasis

## Abstract

**Background:**

Contrary to the advantageous anticancer activities of curcumin (Cur), limited bioavailability and solubility hindered its efficacy. Here, nontoxic dendrosomal nano carrier with Cur was used to overcome these problems. Despite considerable antitumor properties of Oxaliplatin (Oxa), the limiting factors are drug resistance and adverse side-effects. The hypothesis of this study was to evaluate the possible synergism between dendrosomal nanocurcumin (DNC) and Oxa and these agents showed growth regulatory effects on SKOV3 and OVCAR3 cells.

**Methods and materials:**

In the present study, colony formation, wound healing motility, cell adhesion, transwell invasion and migration assay and cell cycle arrest with or without DNC, Oxa and Combination were defined. In addition to, real time PCR and Western blot were used to analyze AKT, PI3K, PKC, JNK, P38 and MMPs mRNAs and proteins expressions. Docking of MMP-2-Cur, MMP-2-DNC and MMP-2-Oxa was performed and the results of all three complexes were simulated by molecular dynamics.

**Results:**

Our findings illustrated that DNC had the greatest effect on cell death as compared to the Cur alone. Moreover, the growth inhibitory effects (such as cell death correlated to apoptosis) were more intense if Oxa was added followed by DNC at 4 h interval. However, insignificant effects were observed upon simultaneous addition of these two agents in both cell lines. Besides, a combination of agents synergistically alters the relative expression of MMP-9.

**Conclusions:**

The docking results showed that His70 and Asp100 may play a key role at the MMP-2 binding site. The matrigel invasion as well as cell viability of ovarian cancer cell lines SKOV3 and OVCAR3 by DNC alone or in combination with Oxa was inhibited significantly. The inhibitory effects of these agents were due to the differential expression levels of MMP 2 and MMP 9 regulated by multiple downstream signaling cascades. From the molecular dynamic simulation studies, it was confirmed that DNC established a strong interaction with MMP-2.

## Introduction

Ovarian cancer is the most frequent cause of death between gynecological cancer, and the fifth cause of cancer mortality among women worldwide. In 2020, worldwide, 313,959 new cases of, and 207,252 deaths from ovarian cancer were estimated [1]. While among gynecological cancers, the greatest rates are related to cervical (less developed countries) and endometrial cancers (more developed countries), the overall poor survival rates are considered for ovarian cancer due to the late diagnostic of this type of cancer. Despite good responses to the first-line chemo-therapy drugs, there is not efficient treatment because 80% of patients eventually progress to advanced stages and 5-year overall survival is only 30%, hence the urgent need to seek improvements globally [2, 3]. Oxaliplatin (Oxa) is a member of the platinum drug that is routinely used to care for patients with advanced ovarian cancer [4]. Unfortunately, most of the recurrent ovarian cancers happened due to platinum resistance, and median survival time of patients with resistant tumors is 6 months, and only 27% of these patients live longer than 12 months [5]. In addition, similarly to other platinum drugs, Oxa is more effective in combination with non-platinum drugs, though some adverse side effects are inevitable [5, 6]. Despite advantageous antitumor properties, dose-limiting toxicity and induction of drug resistance have hindered the efficacy and usefulness of Oxa for clinical purposes. One approach to develop anticancer effect of drugs with fewer drawbacks could be the combination of common chemotherapeutic agents with herbal compounds [7–10]. In this context, plenty of plant-derived substances demonstrated the potential of combination with conventional chemotherapeutics to decrease adverse side effects [11].

Curcumin (Cur) is a natural polyphenolic compound derived from the rhizome of turmeric (Curcuma longa), which has efficacious anticancer properties such as apoptosis induction, cell cycle arrest, inhibition of metastasis, and prevention of angiogenesis and other intercellular functions [12, 13]. Recently, a growing body of documents have been reported that Cur has different effects in a variety of diseases and biological capability against inflammation, oxidative stress, and angiogenesis [14]. However, there are major obstacles for bringing this therapeutic com-pound into the clinic, including poor bioavailability, low aqueous solubility and minor cellular uptake [15]. To overcome these significant -drawbacks, different formulations of Cur have been prepared such as liposomal encapsulation, polymeric nanoparticles, solid dispersions and emulsions [16–18]. In this regard, our group synthesized dendrosome as a safe, biodegradable, and amphipathic nanocarrier. We have demonstrated various studies on the drug delivery capability of dendrosome nanoparticles in different cancers and dendrosomal nanocurcumin (DNC) is one of them [19].

Tumor metastasis begins with cell adhesion of cancer cells, invasion to extracellular matrix (ECM), and migration to other organs. To this purpose, ECM degrades by activation of several enzymes such as matrix metalloproteinase (MMPs). Notably, MMPs (such as MMP-2 (gelatinase A) and MMP-9 (gelatinase B)) have been introduced to degrade type IV collagen to facilitate cancer cell invasion and metastasis in ovarian cancer [20].

Extracellular signal regulated kinase (ERK), p38, and c-Jun N-terminal kinase (JNK) are categorized as major members of the mitogen-activated protein kinase (MAPK) family [21, 22]. The different components of the MAPK intracellular signaling pathway activate each other through a complex network. Disturbance in modulation of MAPK components plays a key role in biological and pathological disorders [23]. Previous studies investigated the expression of some MAPK members in ovarian carcinoma. In this context, activation of MAPK members was re-ported after treatment of ovarian carcinoma cell lines with paclitaxel or cisplatin which significantly affected MAPK levels, proliferation or apoptosis [24]. Although not enough and reliable clinical information exists, exploring a possible correlation between MAPK expression and proliferation and apoptosis markers could be helpful for further clinical studies.

Altogether, in this study we investigated the effects of Oxa, DNC, and their combinations on the viability and other oncogenic properties of OVCAR3 and SKOV3 ovarian cancer cell lines.

## Materials and methods

### Protein–ligand docking

Cur and DNC structures were constructed based on SMILES using the building structure command in Chimera v 1.13.1. Oxa structure was retrieved from PubChem. After energy minimization, all compounds were saved as mol2. The structure of MMP-2 (A-chain) was obtained from RCSB database (1HOV) and was prepared after structure refinement. Based on bioinformatics studies, the catalytic domain A of MMP-2 was selected for docking. MMP-2 binding sites were predicted using the Raptor X server. The MMP-2 structure was prepared for docking by side chain minimization step. The water molecules were removed from MMP-2 and the structure was energetically corrected. The ligands, along with the MMP-2 structure, were introduced into Molegro Virtual Docker v5.5 (MVD) software. The appropriate grid box was then defined at the cavity of the binding site and the ligand–protein docking was run. A run with the lowest binding energy was selected as the best complex. H-Bond formation was demonstrated using LigPlot + software.

### Molecular dynamic simulation

We used Gromacs v 4.6.5 with the CHARMM36 all-atom force field to calculate the molecular dynamics (MD) between the MMP-2-Cur, MMP-2-DNC, and MMP-2-Oxa complexes. The topology of the ligands was obtained from the CGenFF retrieval server (https://cgenff.umaryland.edu, October 30, 2019). The protein–ligand complexes were filled by water molecules. Minimizing the system energy was done after the systems were neutralized. For NVT and NPT factors, optimization was performed at 300 K, 1 bar pressure, and restraint forces of 1000 kJ/mol. All bonds were constrained applying the LINCS algorithm during the simulation. Finally, molecular dynamics simulation after adjustment was performed for 50,000 ps. All of the MD results were visualized by Grace Software in the Linux operating system.

### Chemical agents stock preparation

Oxa and Cur were purchased from Merck Company (Darmstadt, Germany, 95% purity). For the stock solution preparation of Oxa, DMF and mQ water (1:5, v/v) was used as solvent while for Cur stock preparation, ethanol and DMSO was taken as solvent. For dendrosome preparation, PEG400, oleoyl chloride, triethylamine, and chloroform were procured from Sigma-Aldrich Company. The preparation of dendrosome and DNC was as per the previous reports [19, 47–53].

### Cell lines

The ovarian cancer cell lines used in this study (OVCAR3 and SKOV3) were gifted from Dr. Rabbani (Avicenna Research Institute). They were cultured in RPMI 1640 medium (Gibco, NY, USA) supplemented with 10% fetal bovine serum (FBS), 100 U/mL penicillin and 100 µg/mL streptomycin and incubated at 37 °C with 5% CO2.

### Colony formation assay

For this assay, cells were seeded at 500 cells per 6-wells plate and allowed to attach overnight. The cells were treated with lower concentration of IC50 with DNC, Oxa, or a combination of them and cultured under standard conditions at 37 °C and 5% CO_2_ in an incubator. After 10 days, the dishes were washed twice in PBS, fixed with methanol, and stained with crystal violet. After washing the dishes with water and air drying, the number of colonies was calculated and the percent of colonies was defined with the number of colonies formed in treatment divided by the number of colonies formed in control groups without treatment.

### Wound healing motility assay

SKOV-3 cells were seeded in 12-well plates at 4 × 105 cells/well and cultured in a medium containing 10% FBS to near cell monolayer confluency. The scratch wound was created by a p200 pipette tip and washed with PBS to remove cellular debris or the detached cells. Cells were incubated with different treatment conditions (Cur, DNC, Oxa, and a combination) for 0, 6, 12, 24 h in RPMI 1640 serum-free medium at 37 °C as compared to dendrosome and cells without treatment as a negative control. Cell migration into the wound area was measured using the Image J software based on the individual scratch width. The scratch width at 0 h was supposed to be 1 µm) and calculated according to the following equation: [54].

% wound closure = (scratch width in 0 h-remaining scratch width in 24 h)/(scratch width in 0 h) × 100.

### Cell adhesion assay

In this assay, the 96-well plate was coated with 100 μL/well of 50 μg/mL matrigel, 100 μL/well of 20 μg/mL bovine serum albumin (BSA) for 24 h at 4 °C. The nonspecific binding sites were blocked with 0.1% BSA for 1 h at 4 °C. Followed by washing three times with PBS. The SKOV3 and OVCAR3 cells were treated with or without indicated concentration of DNC, Oxa and combination of them for 48 h. The incubated cells were trypsinized and resuspended in 1640 serum-free medium; 3 × 104 cells were added to every prepared well. The cells were incubated at 37 °C for 1 h and the non-adherent cells were removed by three times washing with PBS. The medium was removed, and plates were washed twice in PBS. Cells were then fixed with methanol and stained with 0.1% crystal violet solution, gently washed 3 times with PBS, and crystal violet-solubilized using v/v acetic acid/methanol/water (10:30:60), and absorbance was read at 595 nm.

### Cell invasion assay

The invasive capability of ovarian cancer cells was assayed using Transwell Filter (8 μm pore size, Corning) and Matrigel (BD Biosciences, CA, USA). Briefly, poly vinyl pyrrolidone-free polycarbonate filters (Millipore) (8μ M pore size) were coated with matrigel (15 μg/filter). Briefly, SKOV3 and OVCAR3 cells were treated with lower concentration of IC50 with DNC, Oxa and combination of them for 24 h, then the cells were transferred in each upper chamber in 200 μL of serum-free medium and medium with FBS was used in lower chamber which acts as a chemo attractant. Cells were incubated for 24 h, after incubation, the non-invading cells in the upper chamber were removed. The invaded cells on the lower surface of the membrane were fixed and stained with methanol and crystal violet respectively. Finally, we washed the stained cells, photographed and counted them under a light microscope in at least 10 randomly selected fields.

### Transwell migration assay

A transwell migration assay was done according to the manufacturer’s protocol in 24 well plates similar to invasion assay but the chamber was not coated and only considered the ability of cells to transfer the pore. After treatment cells were incubated in the chamber for 48 h, after incubation, the media in the upper part of the chamber was removed and the migrative cells on the lower surface of the membrane were fixed and stained like an invasion assay.

### Cell-cycle analysis by flow cytometry

To analyze the effect of the experiment on the cell cycle, approximately 1.5 × 105 cells/well of SKOV3 and OVCAR3 were seeded onto six well plates. After 24 h, cells were treated with dendrosome, Cur, DNC, Oxa, and a combination of DNC and Oxa according to proper concentration 3 times separately. Forty-eight hours after treatments, cells were washed, trypsinized, and fixed on ice with 70% cold ethanol and incubated at 20 °C. Then, the cells were washed in PBS and resuspended in a solution of 10 mg/mL propidium iodide (Molecular Probes, Invitrogen, UK) and 0.2 mg/mL RNAase–DNAase free (Sigma, St. Louis, MO, USA) in PBS for 30 min, at room temperature in the dark. The percentages of cells in the different phases of the cell cycle were calculated using a Multi cycle for Windows 64-bit (Beckman Coulter, USA). Gating the cells was done to exclude cell doublets, clumps, and cell debris.

### Evaluation of mRNA expression by semiquantitative RT-PCR

After cell treatments, total RNA was extracted from SKOV3 and OVCAR3 cells using Trizol reagent (Invitrogen Life Technologies; USA) and treated with DNaseI digestion (Thermo Fisher Scientific, Waltham, MA, USA). Five-hundred ng of the total RNA was used for the complementary DNA synthesis by PrimeScript™ RT reagent kit (Takara Bio Inc., Shiga, Japan). After quantification and equalization of the cDNA, real-time PCR was performed with 2µL of cDNA and SYBR green master mix (Biofact, Korea) in a total reaction volume of 10 µL on IQ5 (Bio-Rad, Germany). The sequences of specific primers were demonstrated in Table 1. The changes in gene expression were normalized with GAPDH mRNA expression and the data were analyzed using 2-ΔΔct.

**Table 1:** List of specific primers used in real-time polymerase chain reaction assay

Gene	Designed oligonucleotide	Amplicon length (bp)
	TGGCACCTTCATTGGCTAC	
AKT	CCGCTCCGTCTTCATCAG	106
	GGAACAGCACCTCCACTATTT	
PKC	GCCACAATGTCTGCGTATCT	226
	TGCTGTGTGGAATCAAGCAC	
JNK	CCTCGGGTGCTCTGTAGTAG	168
	ACCATGTTCAGTTCCTTATCTACC	
P38	CTGTCATTTCATCATCTGTGTGC	168
	GGCTCCTCTCCAGTCTGATC	
PI3K	AGCTTAGGAACGTGTGGTCA	200
	TTGATGGCATCGCTCAGATC	
MMP2	TTGTCACGTGGCGTCACAGT	175
	GACGCAGACATCGTCATCCA	
MMP9	CACAACTCGTCATCGTCGAAA	190
	GAGTCAACGGATTTGGTCGT	
GAPDH	TTGATTTTGGAGGGATCTCG	237

### Western blot analysis

For Western blot analysis, 3 × 105 cells were seeded in a 6-well plate and after overnight incubation at 37 °C, cells were treated similarly to another method with dendrosome, Cur, DNC, Oxa and a combination of DNC and Oxa for 48 h. The cells were washed with PBS and resuspended in ice-cold RIPA buffer (25 mM Tris HCl (pH 8.0), 1% Nonidet P40, 0.5% sodium deoxycholate, 0.1% sodium dodecyl sulfate (SDS), and 125 mM NaCl) containing the Complete Protease Inhibitor Cocktail (Roche Diagnostics GmbH, Mannheim, Germany) for 30 min. The ex-tracts were centrifuged at 12,000 rpm at 4 °C for 30 min. Protein concentrations were calculated with Bradford reagent (Sigma Aldrich). Equal concentration of protein (80 μg/lane) were sepa-rated with 12% SDS polyacrylamide gel and transferred onto 0.22 μm poly vinylidene difluoride (PVDF) membrane. To prevent the non-specific binding of antibodies, the membrane was blocked by 5% non-fat milk in Tris buffered saline (TBS) plus 0.1% Tween 20. Subsequently, the membranes were incubated overnight at 4 °C with the following mouse monoclonal anti human primary antibodies: MMP 9 (1:1000; sc 21733; Santa Cruz Biotechnology, Inc.), MMP 2 (1:1000; sc 13594; Santa Cruz Biotechnology, Inc.), and β-actin (1:5000; cat. no. 1025; Cell Signaling Technology, Beverley, MA, USA). Following 3 times washing with TBS-T for 5 min, the membrane was incubated for 1 h at room temperature with a horseradish peroxidase-conjugated secondary antibody after 3 times washing with TBS-T; the membrane was washed with TBS for 1 min and proteins visualized with an ECL detection kit followed by exposure to X-ray film.

### Statistical analysis

Statistics were presented in SPSS 16.0 software and performed using one-way ANOVA analysis of variance followed by Tukey’s HSD multiple comparison test. Differences among groups were statistically significant when *p* < 0.05.

## Result

### Protein–ligand docking

**Fig. 1:**
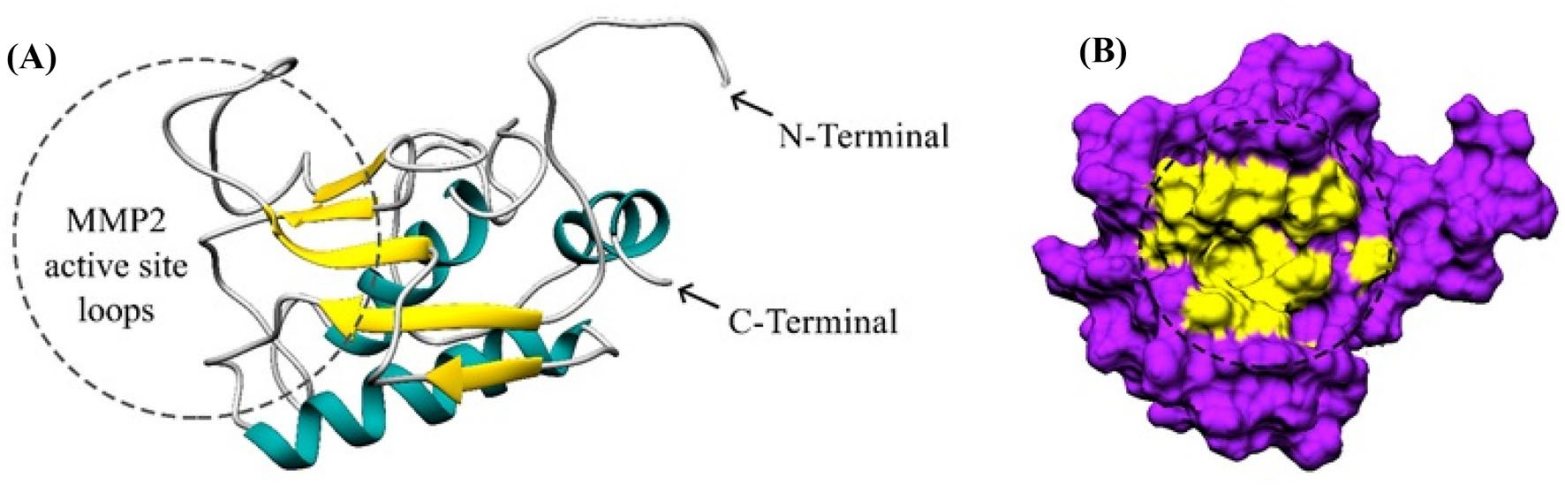
**A** Ribbon diagram of MMP-2 catalytic domain generated from PDB code 1HOV [25]. α-helices, β-sheets, and coils are shown in cyan, yellow, and grey, respectively. **B** Surface structure of MMP-2 catalytic domain where active site is shown in yellow

In this study, we used the crystallographic structure of the MMP-2 (1HOV) to show how Cur, DNC, and Oxa bind to MMP-2. As shown in Fig. 1A, the domain catalytic MMP-2 consists of 3 α-helix and 5 β-sheet. The region containing the amino acids that make up the binding site of the MMP-2 is shown as a surface (Fig. 1B). The results of the RaptorX server deter-mined the potent binding site on the MMP-2 and His70, Gly81, Leu82, Leu83, His85, Ala88, Asp100, Leu116, Val117, His120, Glu121, His124, Glu129, His130, Leu137, Pro140, Ile141, Tyr142, and Thr143 were used as binding pocket residues for docking.

**Fig. 2:**
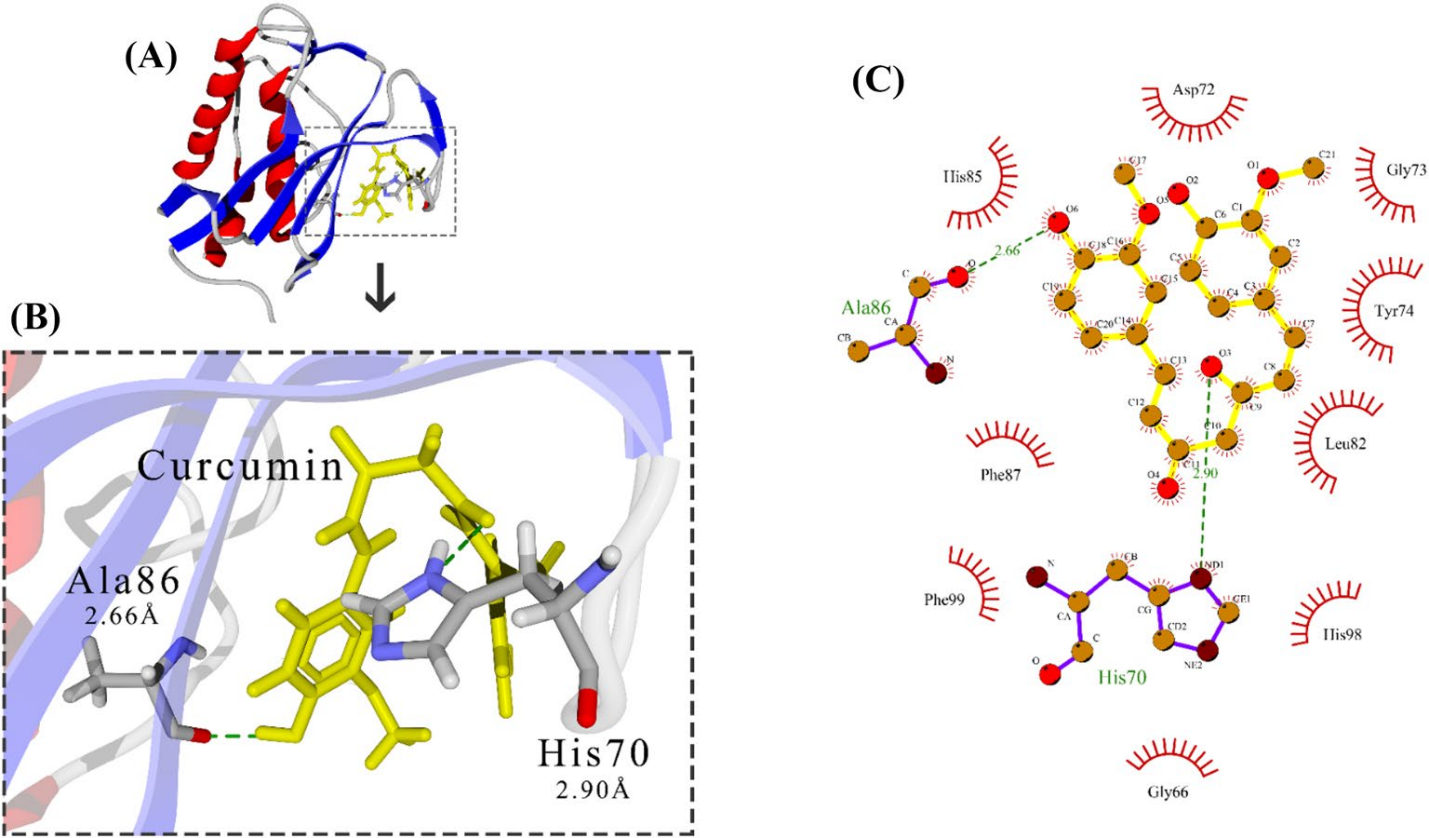
Docking of Cur with MMP-2. **A** MMP-2-Cur complex magnified. **B** MMP-2 structure is shown as ribbon, α-Helix is red, β-sheet is blue, coil is light gray, involved amino acids in complex are element gray, ligand is yellow stick, and H-Bond is dotted line green. **C** LigPlot of the MMP-2-Cur complex. Ligand is yellow, involved amino acids in complex are purple, H-Bonds are dotted line green, and side residues are red

**Fig. 3:**
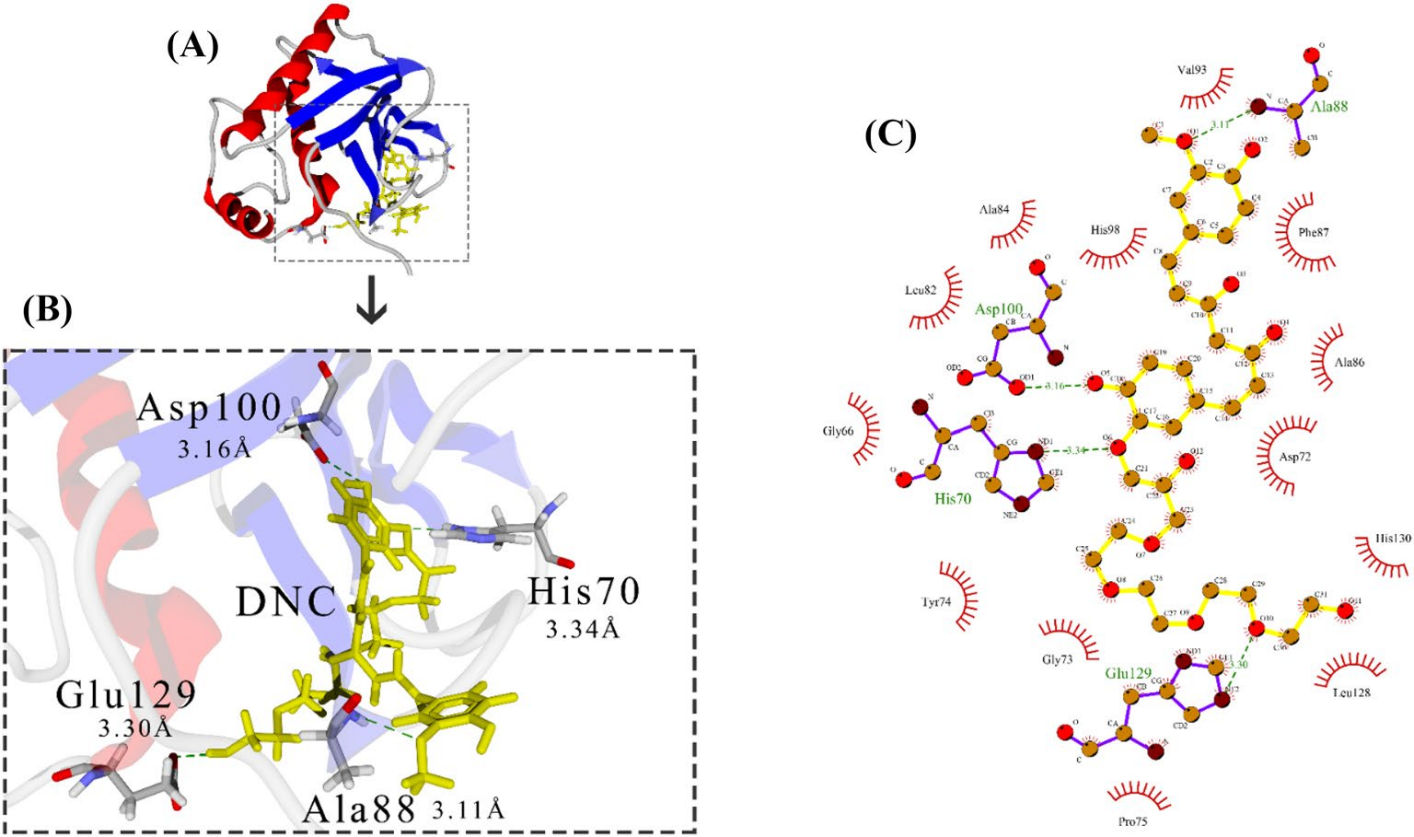
Docking of DNC with MMP-2. **A** MMP-2-DNC complex magnified. **B** MMP-2 structure is shown as ribbon, α-Helix is red, β-sheet is blue, coil is light gray, involved amino acids in complex are element gray, ligand is yellow stick, and H-Bond is dotted line green. **C** LigPlot of the MMP-2-DNC complex. Ligand is yellow, involved amino acids in complex are purple, H-Bonds are dot-ted line green, and side residues are red

**Fig. 4:**
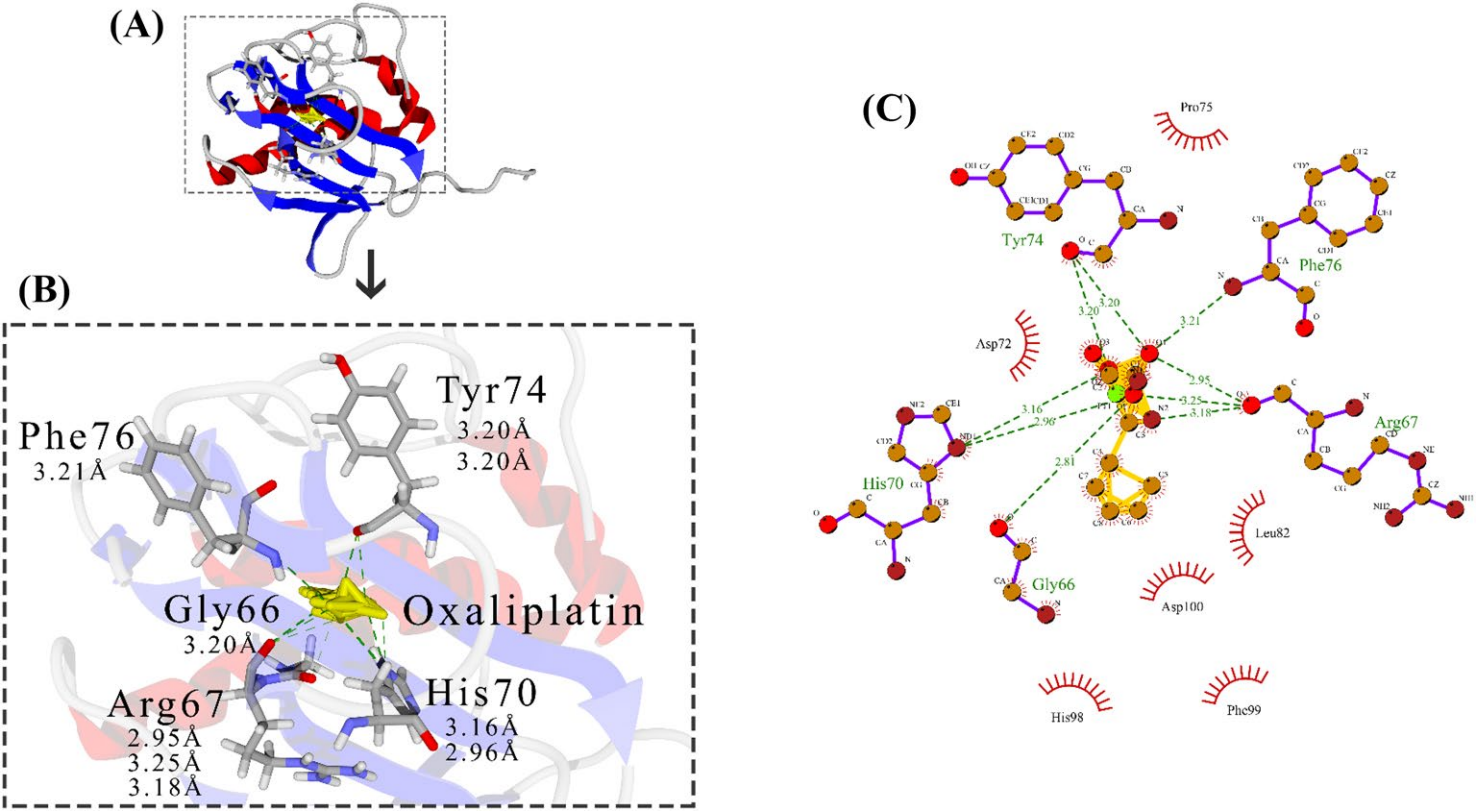
Docking of Oxa with MMP-2. **A** MMP-2-Oxa complex magnified. **B** MMP-2 structure is shown as ribbon, α-Helix is red, β-sheet is blue, Coil is light gray, involved amino acids in complex are element gray, ligand is yellow stick, and H-Bond is dotted line green. **C** LigPlot of the MMP-2-DNC complex. Ligand is yellow, involved amino acids in complex are purple, H-Bonds are dot-ted line green, and side residues are red

We used MVD software for docking protein–ligand. The highest ranking was chosen for each complex with the lowest intermolecular binding energy (Figs. 2A, 3A, 4A). All hydro-gen bonds between MMP-2 and ligands are also shown by magnification. Investigation of the involved amino acids in the MMP-2-Cur complex confirmed that His70 and Ala88 of MMP-2 form H-bonds with Cur at 2.9 and 2.66 Å respectively (Fig. 2B). The MMP-2-DNC complex showed that MMP-2 forms H-bonds with DNC via His70, Ala88, Asp100, and Glu129 (Fig. 3B). It was also observed that in MMP-2, Oxa formed H-bonds with Gly66, Arg67, His70, Tyr74 and Phe76. (Fig. 4B).

Docking results indicate that Cur, DNC, and Oxa compounds can bind to MMP-2 active site residues. His70 jointly interacted with the ligands in all three complexes. Hydrogen bonds were visualized by green dotted line and 2D information on other interactions is shown in Figs. 1C, 2C, 3C.

### Molecular dynamics simulation

**Fig. 5:**
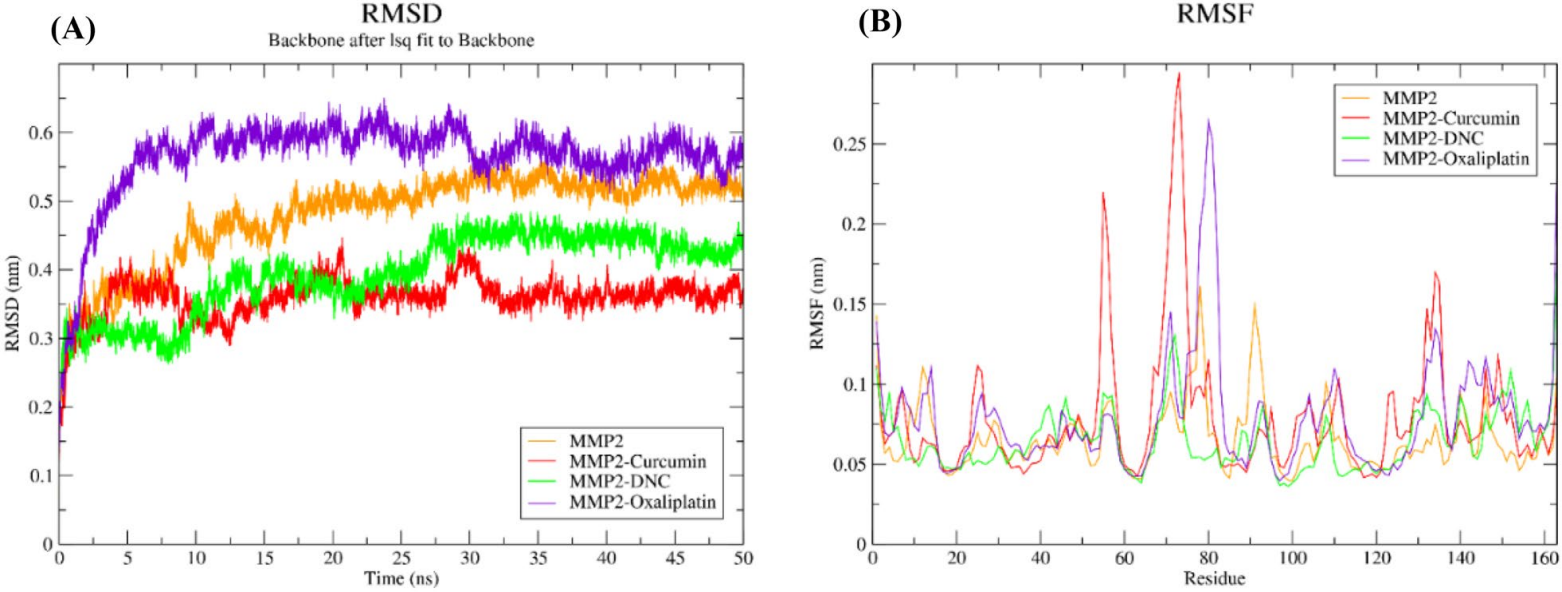
Molecular dynamics simulations of complexes. **A** RMSD and **B** RMSF graph of MMP2 (orange), MMP-2-Cur (red), MMP-2-DNC (green), and MMP-2-Oxa (purple) for 50 ns MD simulation

MD simulations were performed for 50 ns for all complexes. Blank MMP-2 structure was used to control. The MD simulation results showed that the formation of hydrogen bonds in the MM-P2-DNC and MMP-2-Oxa complexes between the ligands and the MMP-2 active site causes the complex to disappear so that root mean square deviations (RMSD), which were at the beginning of the simulation at the highest level, decreased at the end of the simulation (Fig. 5A). These results indicated that DNC and Oxa interacted more strongly with MMP-2 than Cur. Furthermore, the decrease in root mean square fluctuation (RMSF) in MMP-2-DNC and MMP-2-Oxa complexes were lower than in MMP-2-Cur. The RMSF for the MMP-2-DNC and MMP-2-Oxa complexes were about 0.05 nm, while it increased up to 0.15 nm for MMP-2-Cur simulation (Fig. 5B).

### DNC and Oxa inhibited the cell population growth of human ovarian cancer

Since proliferation shows the malignancy of tumors, the similar concentration of DNC, free Cur, and empty dendrosome were used to understand the anti-proliferative effect of them on SKOV3 and OVCAR3 cells with MTT assay. Our previous report [10] showed that DNC could suppress proliferation of these cells more than free Cur significantly in time and dose dependent manner while empty dendrosome had insignificant effect. Similarly, the growth of these cell lines was also inhibited by Oxa in a dose dependent manner. The IC50 values in 48 h for DNC and Oxa treatments in SKOV3 cell line were 22 µM and 25 µg/mL while these values were 15 µM and 12 µg/mL in OVCAR3 [10]. The combination indices (CIs) of these treatments were representative for the synergistic effects [10].

### The impact of DNC, Oxa, and the combination of them on the colony formation

**Fig. 6:**
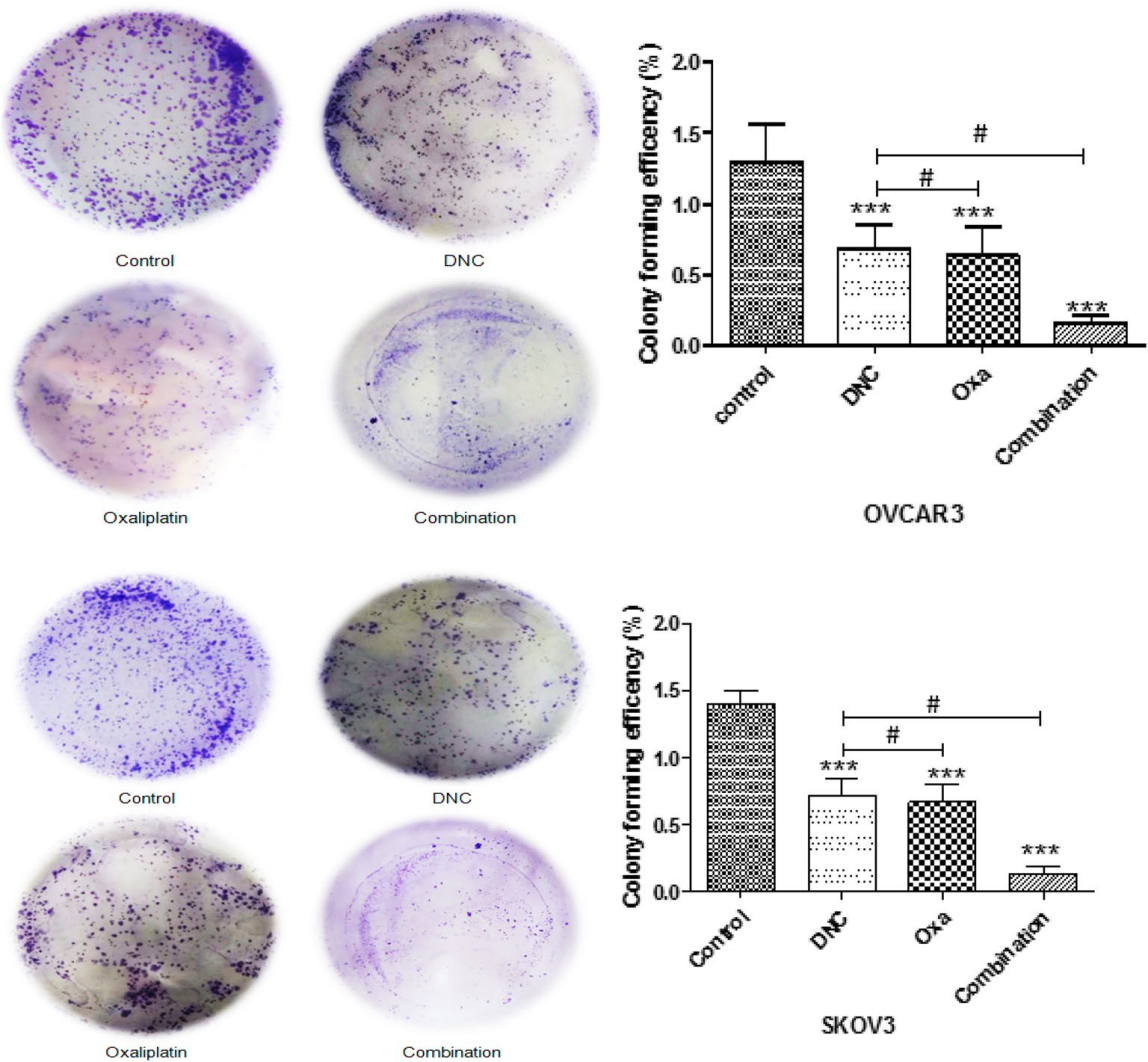
DNC, Oxa, and combination inhibits colony formation of (**A**) OVCAR3 and (**B**) SKOV3 ovarian cancer cell lines. Data show the representative of three independent experiments. Notes: Data expressed as mean ± standard deviation; *** p < 0.001 compared to no treated cells and # p < 0.05 combination compared to DNC and Oxa

A colony formation assay demonstrated that the colony numbers of the SKOV3 and OVCAR3 cells treated with DNC, Oxa, and the combination of them were significantly decreased compared with the control group (p < 0.001; Fig. 6A, B). The inhibitory effect of the combination group was significantly more than the DNC or Oxa treatment group in two cell lines.

### DNC and Oxa inhibited the motility of SKOV3 cells

**Fig. 7:**
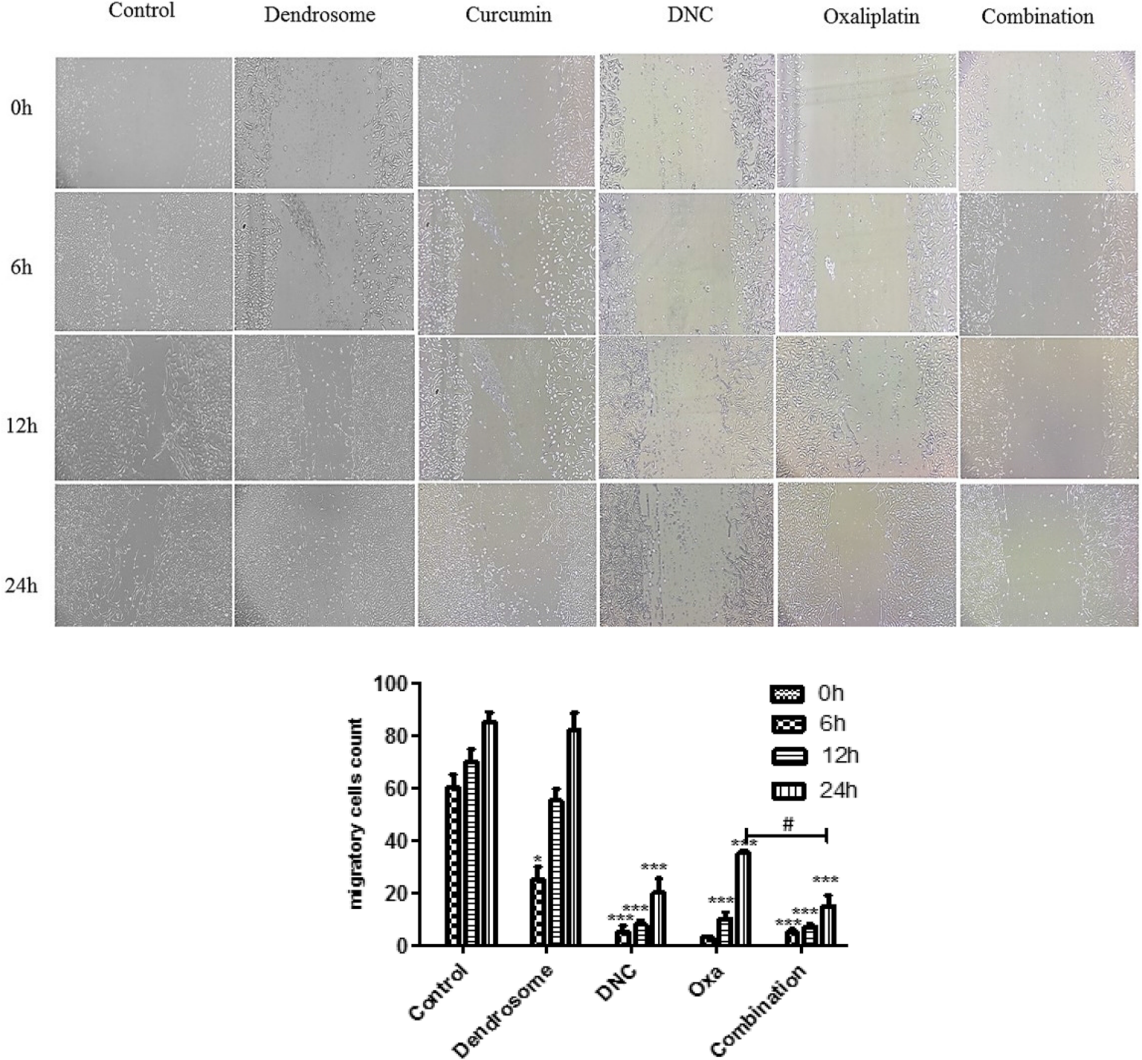
DNC, Oxa, and Combination inhibit the motility of SKOV-3 cells in a dose-dependent manner. **A** Untreated and treated wound scratches in 12-well plate at different time intervals. **B** Migratory cell counts in the untreated and treated scratch area. Notes: Data expressed as mean ± standard deviation; * p < 0.05; *** p < 0.001 compared to no treated cells in every time point and # p < 0.05 combination compared to Oxa

Visible view of microscopic image showed that scratch width of the cell-free zone was de-creased from 0 h until 24 h in all of the treatments but the rate of migrated cells after treatment was significantly less than the negative control and dendrosome. Furthermore, the migration rate of the combination of DNC and Oxa was less than DNC and Oxa alone. The results demonstrated that combination treatment significantly inhibits the motility of SKOV3 cells (Fig. 7).

### Inhibition of SKOV3 and OVCAR3 cells adhesion

**Fig. 8:**
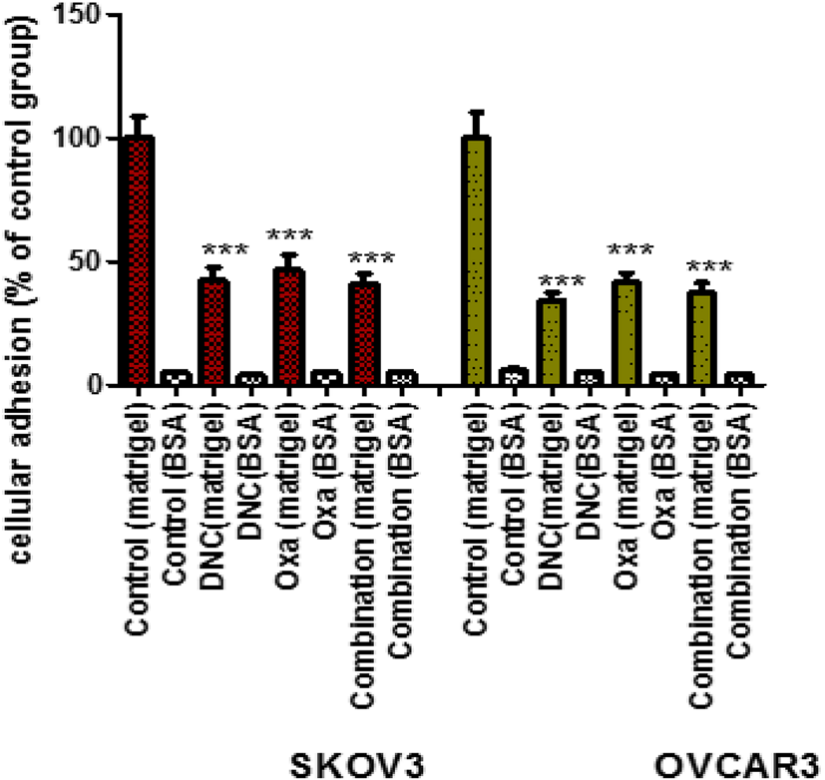
Effects of DNC, Oxa and Combination treatments on the cell adhesion of SKOV3 and OVCAR3 ovarian cancer cells. The data represent *** p < 0.001 vs. control group

The first step of tumoral cell invasion is adhesion of cancer cells to extracellular matrix (ECM). Cell adhesion assay was done to define whether our treatment reduced SKOV3 and OVCAR3 cells adhesion to matrigel and BSA. We found all of the treatments significantly de-creased cell attachment to matrigel compared with the control group, while there was no significant reduction between combination and single agent treatment in two cell lines (Fig. 8).

### Inhibitory effect of DNC, oxa, and combination therapy on invasion and migration of cells

**Fig. 9:**
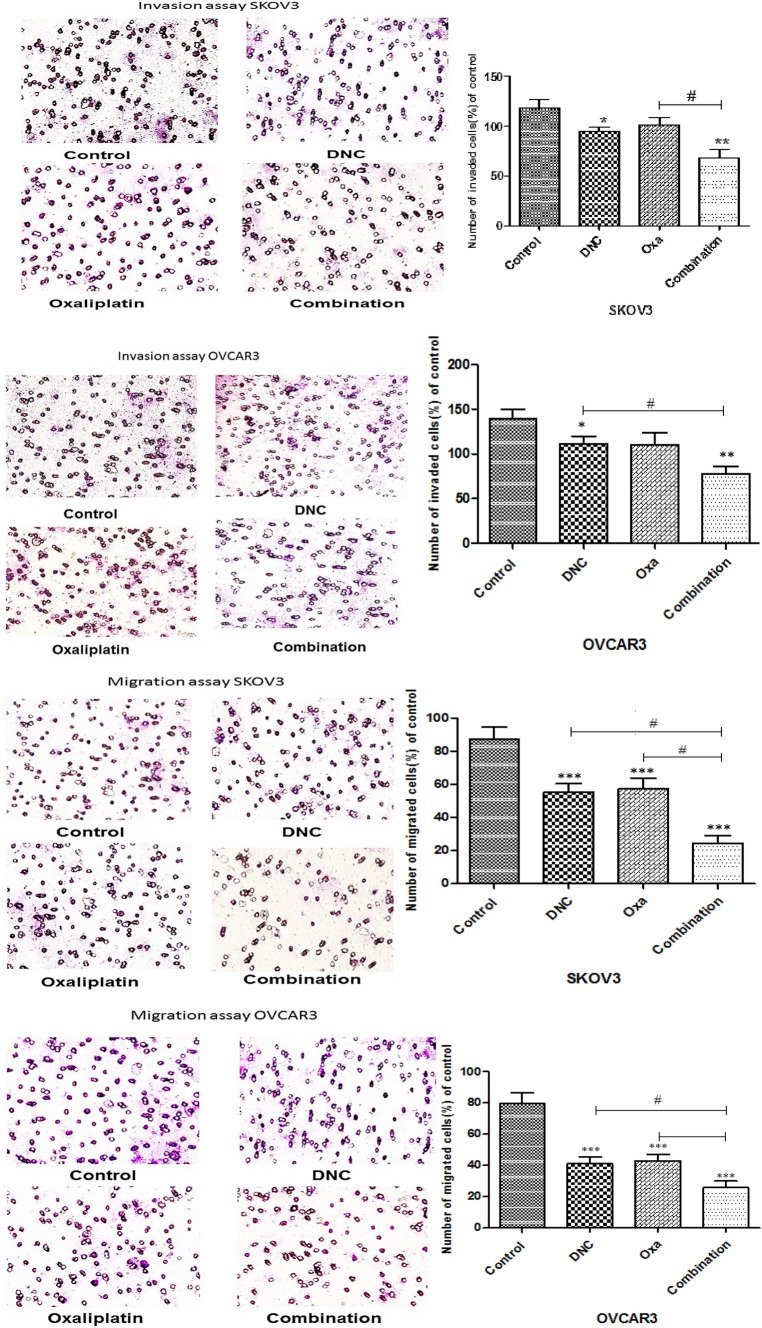
DNC, Oxa, and Combination treatments suppressed the invasion (**A**, **B**) and migration (**C**, **D**) of SKOV-3 and OVCAR3 cells. Notes: Data expressed as mean ± standard deviation; * p < 0.05; ** p < 0.01; *** p < 0.001 compared to no treated cells and # p < 0.05 combination compared to DNC and Oxa

Depending on the migration and invasion phase of metastasis, cancer cells should go over through the ECM. To consider the ability of DNC and Oxa compared with the combination of them, we evaluated the migratory and invasive abilities of the cells by using matrigel-coated and non-coated membranes. As shown in Fig. 9, all of the treatments decreased the invasion and migration of SKOV3 and OVCAR3 cells; while this reduction in the two cell lines was significant in combination compared with the single treatments.

### Change in DNA content of ovarian cancer cells induced by DNC, oxa, and combination treatments

**Fig. 10:**
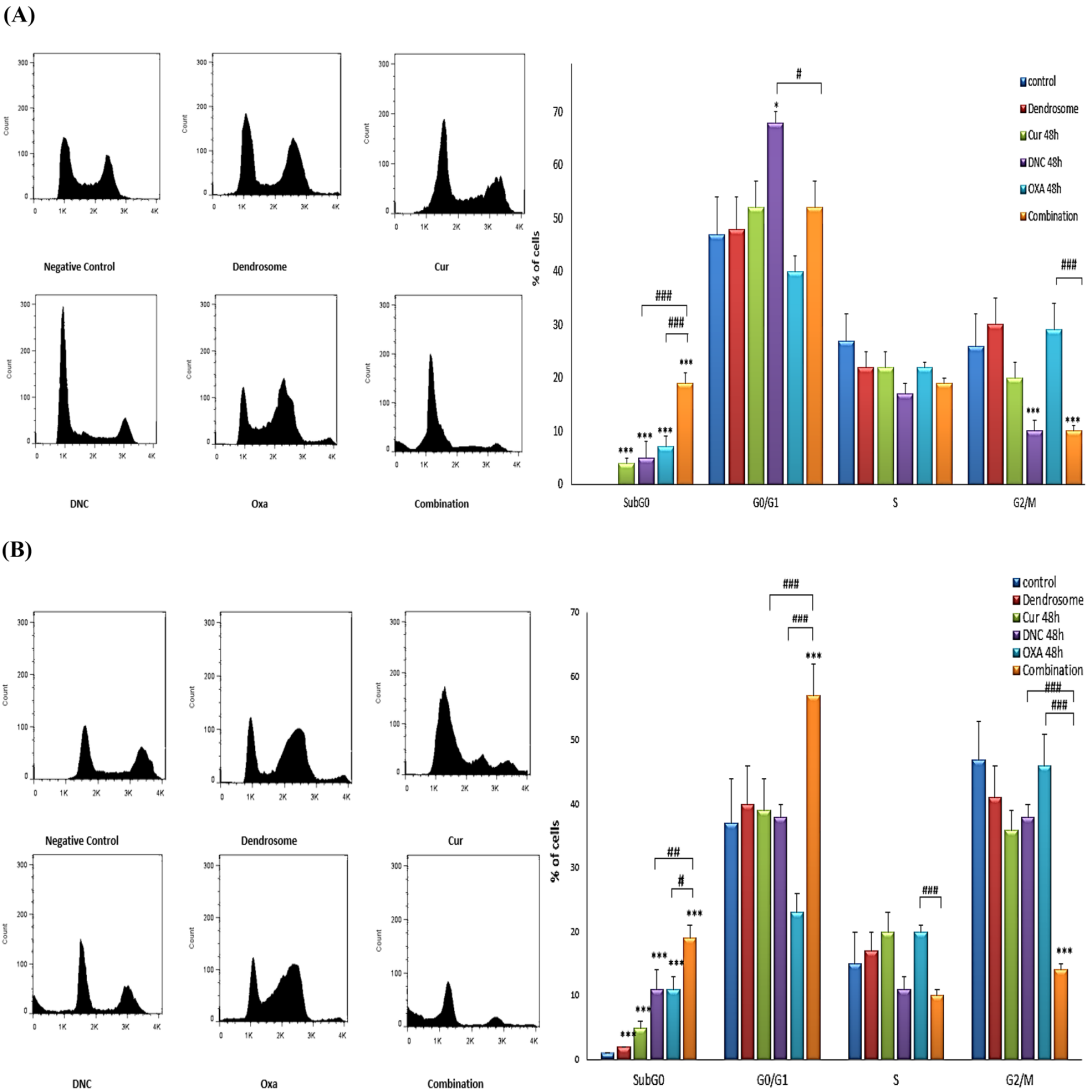
Flow cytometric analysis of the cell cycle in (**A**) SKOV3 (**B**) OVCAR3 cells after treatment with Cur, DNC, Oxa, and Combination of DNC and Oxa. Notes: Data expressed as mean ± standard deviation; * p < 0.05; *** p < 0.001 compared to no treated cells and # p < 0.05; ## p < 0.01; ### p < 0.001 combination compared to DNC and Oxa

The effect of treatments on cell-cycle progression were analyzed by flow cytometry in ovarian cancer cells. In our previous study [10], we investigated the effect of serial dilution of DNC and Oxa in 48 h and demonstrated IC20% of DNC and Oxa as 5 µM and 4.5 µg, respectively, in OVCAR3 and 10 µM and 10 µg in SKOV3. The purpose of this study was to investigate the combination effect of DNC with Oxa at concentrations below IC50 in order to decrease resistance to chemotherapy and to increase sensitivity for the treatments. In cell cycle analysis, we couldn’t see any significant difference between two treated cell lines. Compared to the controls (cells without treatment and with dendrosome), the percentage of cells in G2/M reduced significantly upon combination of DNC and Oxa. In both of the treated cell lines, a progressive accumulation of cell numbers in the sub-G0 phase was observed and this differential change was very significant in the treatments of combination agents compared to the single treatments (*p* < 0.0001) (Fig. 10). In accordance with our previous studies on apoptosis [10], here, the combination treatment exhibited more SubG0 DNA content which can be correlated with a high apoptotic index because cells with DNA content lower (in SubG0) than G0/G1 phase are usually considered as apoptotic cells.

### Change in gene expression by DNC, oxa and combination

**Fig. 11:**
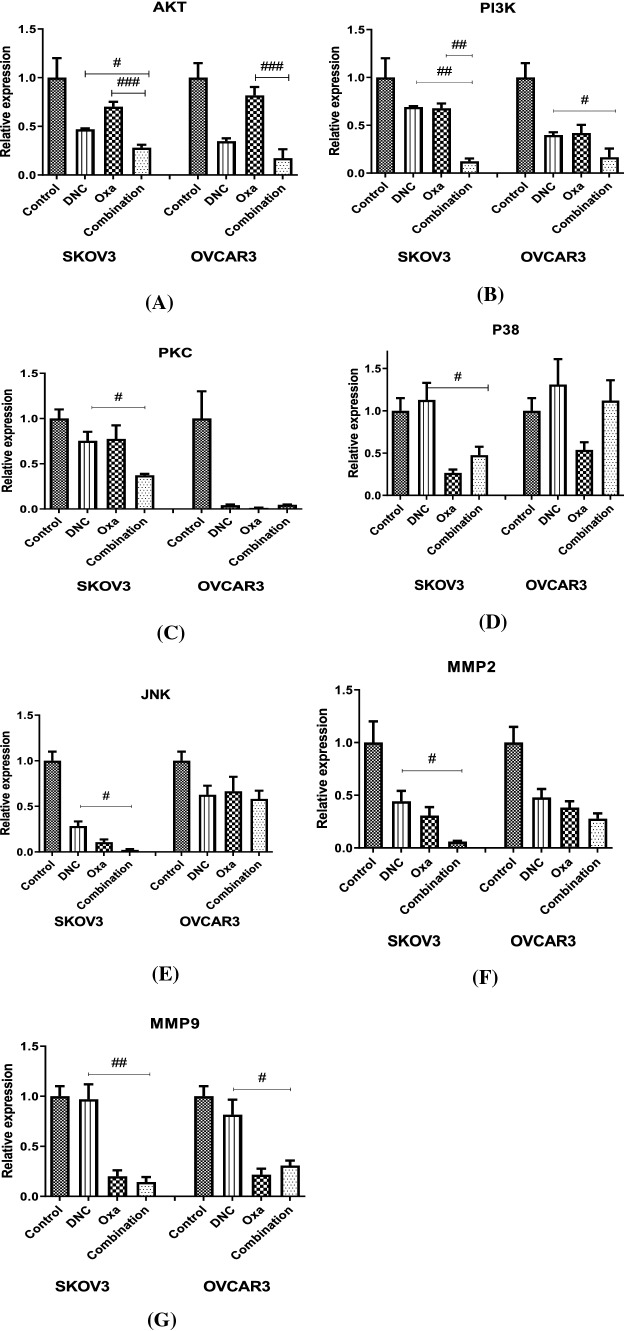
Gene expression analysis in SKOV3 and OVCAR3 cell lines after treatment with DNC, Oxa, and Combination for (**A**) Akt, (**B**) PI3K, (**C**) PKC, (**D**) P38, (**E**) JNK, (**F**) MMP-2 and (**G**) MMP-9. Notes: Data expressed as mean ± standard deviation; # p < 0.05; ## p < 0.01; ### p < 0.001 combination compared to DNC and Oxa

The differential expression of mRNA levels for AKT, PI3K, MAPK1, P38, JNK1/2, MMP2, and MMP9 was observed in the presence of single and combination treatments of DNC and Oxa. In 48 h, the treatments caused a significant reduction in mRNA levels. Mostly, the effect on gene expression was greater in combination rather than in single treatment, except for P38 in OVCAR3 cell line (Fig. 11).

### The decreasing effect of DNC, oxa, and combination treatments on MMP-2 and MMP-9 mRNA expression levels

MMPs are responsible for ECM degradation and consequently, cell invasion [20]. In accordance with the previous reports of MMP expression for the progression of the ovarian cancer, we have tested the effect of DNC, Oxa, and its combination on the differential expression of MMP-2 and MMP-9. It is evident from our result that all the treatments of DNC and Oxa significantly affected MMP-2 expression in both OVCAR3 and SKOV3 cell lines. The change in relative expression level was more prominent in combination treatment than in single treatments of these agents (Fig. 11F). In the case of MMP-9 expression, Oxa treatment was very much significant in both of the cell lines while combination treatment was more prominent in SKOV3. DNC had little in OVCAR3 and there was no effect in SKOV3 cell line (Fig. 11G).

### DNC, oxa, and combination treatments suppressed protein expression of MMP 2 and MMP 9

**Fig. 12:**
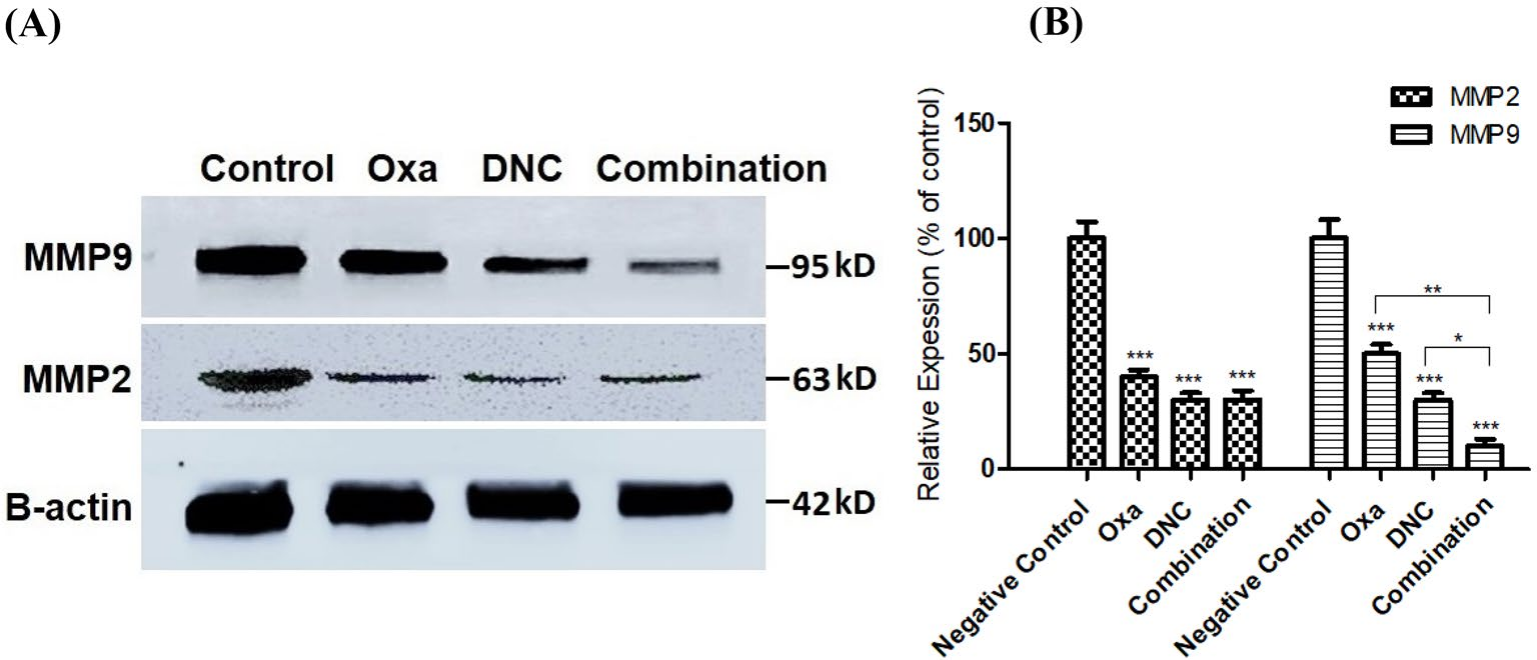
DNC, Oxa, and Combination treatments decrease the expression of MMP 2 and MMP 9 proteins in OVCAR3 cells. **A** The cells were treated with DNC, Oxa, and Combination for 48 h and then applied to Western blotting to analyze the protein levels of MMP 2 and MMP 9. **B** Measurement of the protein levels of MMP 2 and MMP 9 in OVCAR3 cells. Data represent the means ± standard deviation of three independent experiments performed in triplicate. * *p* < 0.05 and ** *p* < 0.01 *** *p* < 0.001 compared with control group and single treatments

MMPs are important marker to cell invasion, so the effect of DNC, Oxa, and its combination on the expression levels of MMP 2 and MMP-9 proteins in OVCAR3 cells was quantified by Western blotting. Figure 12A, B revealed that all the treatments significantly reduced the protein levels of MMP 2 and MMP 9 (*p* < 0.001) while this difference was more significant in combination compared to single agent treatment in MMP-9 protein expression level (*p* < 0.05 and *p* < 0.01).

## Discussion

**Fig. 13:**
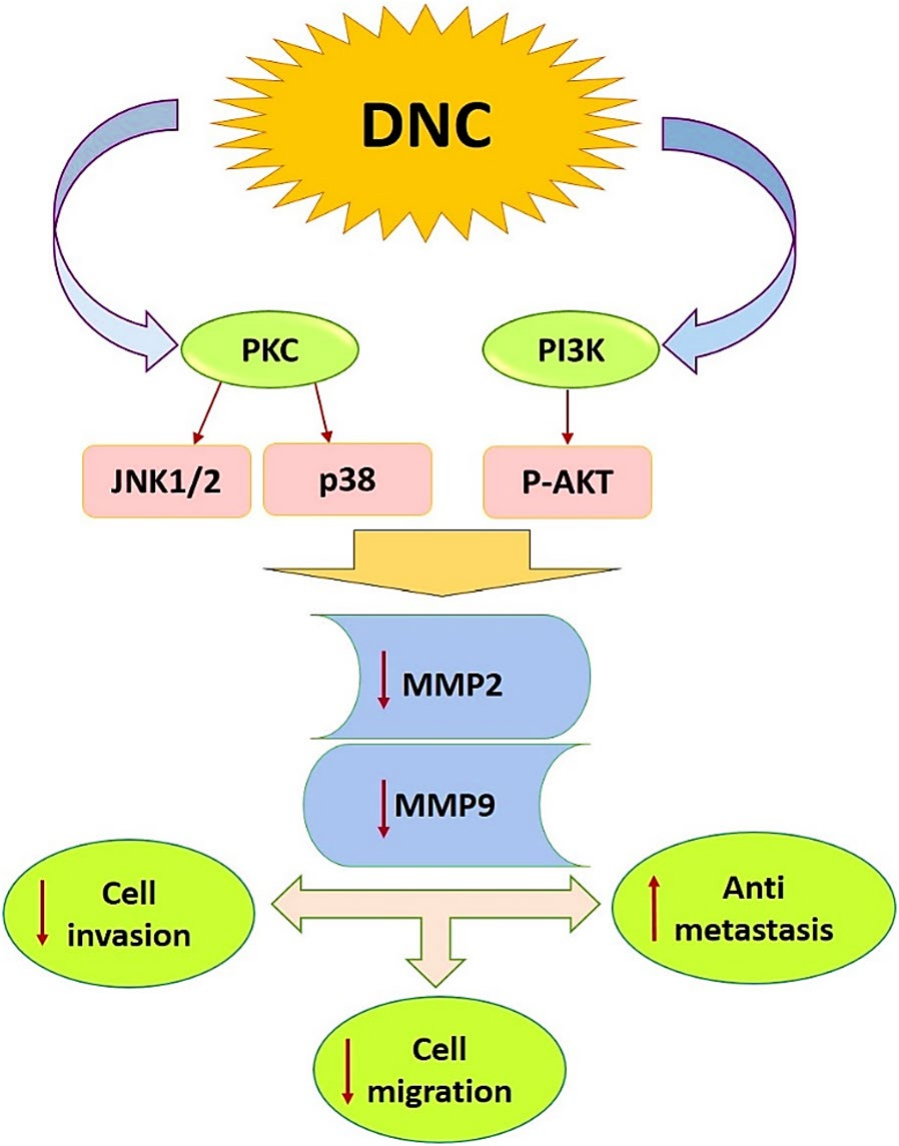
Schematic representation of the effect of dendrosomal nanocurcumin (DNC) on the ovarian cancer call lines by reducing the expression of metalloproteinase (MMP-2 and MMP-9). The bold arrows next to MMP-2 and MMP-9 expression, cell invasion and cell migration are showing downregulation while next to antimetastasis is for upregulation of the respective properties

MMPs are a group of zinc-containing endopeptidases having the potential for degrading extracellular matrix proteins and they thereby play a pivotal role in the metastasis process in different kinds of human malignancies. Because of the widespread physiological importance of MMP-2, it is important to understand how its amino acids interact and play a critical role in MMP-2 activity. Understanding the behavioral mechanisms of MMP-2 can provide new insights into the selection of inhibitors that selectively and specifically inhibit them [26]. Extracellular MMPs often consist of a propeptide, a catalytic domain, and a hemopexin-like domain. In general, MMP-2 is a gelatinase that belongs to the group of metallo-serine proteases [25]. In this study, we used the structure of the MMP-2 catalytic domain. Docking results showed that all three compounds of Cur, DNC, and Oxa interact with MMP-2. Cur interacts with only two MMP-2 amino acids, while DNC and Oxa interact with more MMP-2 amino acids. However, all three compounds interacted with His70. As previously reported, metalloproteases use the histi-dine/aspartate or serine catalytic triad in their active site [27]. In metallo-serine proteases, histi-dine is a proton donor, and the nucleophilic attack is performed by aspartate or serine [28]. Interaction with histidine70 indicates blocking of at least one amino acid in the active site of the MMP-2 by all three compounds. DNC also forms H-bonds with aspartate, glutamine, and ala-nine. In addition to this, Oxa interacts with more amino acids than Cur. The aspartate, alanine, and glutamine are conserved in most MMPs [25]. This indicates that blocking these amino acids can impair MMP-2 function. Ahmad et al. also showed in their studies that Cur interacts with MMP-2 [29]. Docking results were simulated by MD. The decrease in MMP-2-Cur complex RMSD relative to the control (MMP-2) in the analysis of MD results indicates that Cur interacted with the MMP-2 protein. However, RMDS is less stable. MMP-2-DNC complex also has a strong-er interaction with the MMP-2 structure because from 25 ns onwards, the RMSD is almost fixed. MMP-2-Oxa complex alone was able to establish strong interaction with MMP-2 because the RMSD remained almost constant during the simulation. To date, several reports have investigated the effects of Cur on myosin, lipid bilayers, human SOD1 mutant, and Amy-loid-β fibrils by molecular dynamics simulations, respectively [30–33]. However, DNC and Oxa, in cooperation with each other, seem to follow the rule of synergistic effect on MMP-2. The re-sults of the RMSF graph show that the amino acids in the active site of the MMP-2 are more disturbed than the other amino acids because the amount of RMSF in the catalytic loop region (from His70 to Glu129) is increased. In the presence of Cur, DNC, and Oxa RMSF was increased by more than 0.75 nm in His70, suggesting the increase in binding energy between the ligand and the amino acids at the active site of MMP-2. In general, the results of docking and MD simulation show that the combined effect of DNC and Oxa on MMP-2 is greater than that of Cur. Our findings revealed that DNC attenuated the proliferation and viability of ovarian cancer cells in a concentration and time-dependent manner. Moreover, the synergistic effect of DNC with Oxa was verified in our experiments at the concentrations lower than IC50. We also demonstrated that DNC had the potential to sensitize ovarian cancer cells to Oxa treatment synergistically. Notably, our findings are consistent with the results of previous studies indicating that Cur in combination with chemotherapeutic agents including cisplatin, Oxa and cyclophosphamide exerted greater Antiproliferative effects in different cancers [34–36]. We further confirmed the effects of DNC, Oxa, as well as the combination of these two agents on long-term survival of ovarian cancer cells. Our data indicated that DNC and Oxa treatments caused a significant reduction in the colony-forming ability. As expected, the combination of both agents synergistically decreases the number of colonies in both cell lines as compared to single agent treatments. Several intricate processes, including cell migration and invasion, as well as cell adhesion, contributed in tumor metastasis, suggesting the potential therapeutic approaches which are necessary for targeting of degradation of the extracellular matrix [20]. In the present study, we found that combined treatment of DNC and Oxa significantly reduced cell attachment to matrigel compared to the single-agent treatment. However, no significant difference was determined regarding adhesive behaviors of cells with concurrent treatment of both agents. Regarding the migration assay, we figured out that both DNC and Oxa declined the percentage of migrated cells in ovarian cancer cells. Additionally, the combination of two agents caused much more effective reduction in the rate of migration ability. On the flipside, treatment with DNC and Oxa successfully decreases the proportion of invasiveness in both surveyed cell lines, whereas a synergistic effect was identified just in the case of simultaneous treatment of DNC and Oxa in two cell lines. Alteration in the pattern of cell-cycle progression is a fundamental feature of cancer cells as well as development of novel anticancer agents related to the cell cycle arrest and apoptosis. As previous studies rep-resented, Cur has a considerable role in the alteration of cell-cycle process when combined with platinum drugs [36]. Our findings also clearly illustrated that IC20 of both agents through 48 h significantly dwindled the percentage of G2/M phase and increased the level of sub-G1 in SKOV3 and OVCAR3 cell lines. Besides, simultaneous treatment of DNC with Oxa makes ovarian cancer cells more susceptible to the action of Oxa. It has been reported that MMP 2 and MMP 9 are mostly linked with cell migratory and invasive behavior of cancer cells in a variety of tumors [37–39]. A growing body of evidence has demonstrated that the expression level of MMP 2 and MMP 9 in several cancer types is regulated by multiple signaling pathways, includ-ing the wnt/β catenin pathway, and the hedgehog signaling pathway, the phosphoinositide-3 ki-nase (PI3K)/AKT pathway, and the mitogen-activated protein kinase (MAPK) pathway [40–42]. As reported in previous studies, Cur possesses high anti-cancerous characteristics and invasion reduction in different cancers which includes melanoma, prostate, and breast cancers [43–46]. In accordance with these evidences, our current study defined the anti-oncogenic role of nanocurcumin along with oxaliplatin synergistically by inhibiting cell invasion as well as cell migration in ovarian cancer cell lines (Table 2 and Fig. 13).

**Table 2:** Relative effect (%) of the single and combination treatments of Oxa and DNC on the oncogenic. properties of ovarian cancer cell lines

Properties	Cell lines	Treatments		
		Oxa	DNC	Combination
Colony formation	SKOV3	41	37.9	6.8
	OVCAR3	46	42	9.2
Cell motility	SKOV3	40	20	15
Cell adhesion	SKOV3	45	50	42
	OVCAR3	30	42	40
Matrigel invasion	SKOV3	72	80	60
	OVCAR3	67	75	57
Transwell migration	SKOV3	68	70	29
	OVCAR3	50	56	27
Cells in SubG_0_	SKOV3	5	7	20
	OVCAR3	10	10	20
mRNA level: MMP-2	SKOV3	40	25	8
	OVCAR3	50	35	20
mRNA level: MMP-9	SKOV3	100	20	15
	OVCAR3	85	25	32
Protein level: MMP-2	OVCAR3	15	20	15
Protein level: MMP-9	OVCAR3	30	50	10

## Conclusions

The present study investigated the effect of DNC, Oxa, and combination treatments of these two agents on the mRNA and protein expression levels of MMP-2 and MMP-9, as two documented MMPs correlated with ovarian cancer progression. We additionally explored if these two MMPs were modulated through other proteins in the MAPK pathway including PKC, JNK, P38, PI3K, and AKT. Our real-time PCR analysis implied that DNC and Oxa significantly down-regulated the expression level of almost all the surveyed genes in SKOV3 and OVCAR3 except for P38, which was not downregulated by DNC. As expected, combinational administration of both agents had the most obvious down-expression in MMP-2 and MMP-9. However, in the case of P38 and MMP-9 mRNA levels, no synergistic effect was identified in these two cell lines, since the effect of mere Oxa declined the level of both MMPs compared to combination treatment. To clarify the effect of DNC, Oxa, and combination treatments at protein level, therefore, we also performed Western blot analysis. Our data represented that DNC combined with Oxa had much greater effect in downregulation MMP-9 protein expression, whereas such a synergistic effect was not observed in the case of MMP-2.

## Data Availability

All the authors confirm the availability of data and materials.

## Abbreviations

DNC: Dendrosomal nanocurcumin
Oxa: Oxaliplatin
Cur: Curcumin
DMF: Dimethyl formamide
DMSO: Dimethyl sulfoxide
NMR: Nuclear magnetic resonance
FBS: Fetal bovine serum
Pen/Strep: Penicillin/streptomycin
MTT: 3-(4,5-Dimethylthiazole-yl)-2,5-diphenyltetrazolium bromide
IC50: Inhibitory concentration of 50% of cells
MMP: Matrix metalloproteinase
MAPK: Mitogen-activated protein kinase
ERK: Extracellular signal regulated kinase
JNK: C-Jun N-terminal kinase
PVDF: Poly vinylidene difluoride
ECM: Extracellular matrix
PI3K: Phosphoinositide 3-kinase
GAPDH: Glyceraldehyde 3-phosphate dehydrogenase
TBS: Tris buffered saline
ECL: Enhanced chemiluminescence
